# The Effects of MyChoices and LYNX Mobile Apps on HIV Testing and Pre-Exposure Prophylaxis Use by Young US Sexual Minority Men: Results From a National Randomized Controlled Trial

**DOI:** 10.2196/63428

**Published:** 2025-02-05

**Authors:** Katie B Biello, Kenneth H Mayer, Hyman Scott, Pablo K Valente, Jonathan Hill-Rorie, Susan Buchbinder, Lucinda Ackah-Toffey, Patrick S Sullivan, Lisa Hightow-Weidman, Albert Y Liu

**Affiliations:** 1 Department of Epidemiology School of Public Health Brown University Providence, RI United States; 2 The Fenway Institute Fenway Health Boston, MA United States; 3 Department of Medicine Beth Israel Deaconess Medical Center/Harvard Medical School Boston, MA United States; 4 Bridge HIV San Francisco Department of Public Health San Francisco, CA United States; 5 Department of Medicine University of California, San Francisco San Francisco, CA United States; 6 University of Connecticut Waterbury, CT United States; 7 Rollins School of Global Public Health Emory University Atlanta, GA United States; 8 Institute on Digital Health and Innovation College of Nursing Florida State University Tallahassee, FL United States

**Keywords:** HIV testing, adolescents, sexual minority men, mobile apps, pre-exposure prophylaxis, youths, randomized controlled trial, sexual minority, United States, efficacy, LYNX, MyChoices, sociodemographic, behavioral health, HIV prevention, HIV infection, HIV care, transmission, public health, mHealth, mobile phones

## Abstract

**Background:**

Young sexual minority men have among the highest rates of HIV in the United States; yet, the use of evidence-based prevention strategies, including routine HIV testing and pre-exposure prophylaxis (PrEP), remains low. Mobile apps have enormous potential to increase HIV testing and PrEP use among young sexual minority men.

**Objective:**

This study aims to assess the efficacy of 2 theory- and community-informed mobile apps—LYNX (APT Mobility) and MyChoices (Keymind)—to improve HIV testing and PrEP initiation among young sexual minority men.

**Methods:**

Between October 2019 and May 2022, we implemented a 3-arm, parallel randomized controlled trial in 9 US cities to test the efficacy of the LYNX and MyChoices apps against standard of care (SOC) among young sexual minority men (aged 15-29 years) reporting anal sex with cisgender male or transgender female in the last 12 months. Randomization was 1:1:1 and was stratified by site and participant age; there was no masking. The co-primary outcomes were self-reported HIV testing and PrEP initiation over 6 months of follow-up.

**Results:**

A total of 381 young sexual minority men were randomized. The mean age was 22 (SD 3.2) years. Nearly one-fifth were Black, non-Hispanic (n=67, 18%), Hispanic or Latino men (n=67, 18%), and 60% identified as gay (n=228). In total, 200 (53%) participants resided in the Southern United States. At baseline, participants self-reported the following: 29% (n=110) had never had an HIV test and 85% (n=324) had never used PrEP. Sociodemographic and behavioral characteristics did not differ by study arm. Compared to SOC (n=72, 59%), participants randomized to MyChoices (n=87, 74%; *P*=.01) were more likely to have received at least 1 HIV test over 6 months of follow-up; those randomized to LYNX also had a higher proportion of testing (n=80, 70%) but it did not reach the a priori threshold for statistical significance (*P*=.08). Participants in both MyChoices (n=23, 21%) and LYNX (n=21, 20%) arms had higher rates of starting PrEP compared to SOC (n=19, 16%), yet these differences were not statistically significant (*P*=.52).

**Conclusions:**

In addition to facilitating earlier treatment among those who become aware of their HIV status, given the ubiquity of mobile apps and modest resources required to scale this intervention, a 25% relative increase in HIV testing among young sexual minority men, as seen in this study, could meaningfully reduce HIV incidence in the United States.

**Trial Registration:**

ClinicalTrials.gov NCT03965221; https://clinicaltrials.gov/study/NCT03965221

## Introduction

Sexual minority men (eg, gay, bisexual, and other men who have sex with men) account for nearly two-thirds of all people living with HIV in the United States while comprising less than 10% of the population. Sexually active young sexual minority men, aged 13-25 and 25-34 years, have among the highest rates of HIV [1,2]. Young sexual minority men are also the least likely to be aware of their HIV status, further enhancing HIV transmission risks and delaying HIV care [2].

The Centers for Disease Control and Prevention (CDC) recommends all sexually active sexual minority men get tested for HIV at least annually, and consider testing more often (ie, every 3 to 6 months) for sexual minority men who are sexually active [3]. HIV testing is essential for early detection, early care engagement, and early treatment—resulting in a lower likelihood of onward transmission and improved quality of life [3]. For those who test negative, it is also an opportunity to engage in pre-exposure prophylaxis (PrEP) care. The US Preventive Services Task Force expanded the recommendation for PrEP in August 2023 to include adolescents, in addition to adults, who report anal or vaginal sexual activity and disclose one of the following criteria: sexual partner with HIV, a recent bacterial sexually transmitted infection (STI), or inconsistent or no condom use with sexual partners [4]. Despite the efficacy of and need for these evidence-based prevention strategies—routine HIV testing and PrEP—use remains suboptimal among young sexual minority men [5-7].

Reasons for a lack of routine HIV testing and limited PrEP use among young sexual minority men are multilevel and complex [8]. For example, at the individual level, young sexual minority men tend to underestimate their personal risk of acquiring HIV [9,10]; at the interpersonal level, young sexual minority men may be concerned that their partner will feel threatened if they test for HIV or initiate PrEP [11,12]; at the clinical level, young sexual minority men may not be comfortable disclosing their sexual activity to providers [13-15]; at the structural level, young sexual minority men may not know where to access HIV testing or PrEP, or have the autonomy or ability to access a testing site or PrEP clinic on their own [11,16]. Any intervention or program to increase routine HIV testing and PrEP use will need to address a plethora of multilevel challenges [8].

Mobile apps—accessed via smartphones which have nearly complete coverage in the United States, particularly among youths—have enormous potential to increase routine HIV testing and PrEP use among young sexual minority men [17-19]. Ninety-five percent of youth report having a smartphone [20], and youth commonly use their mobile devices for a range of activities, including downloading health-related apps and accessing health information [21]. Implemented through mobile apps, mobile health (mHealth) interventions may be a promising tool for promoting behaviors such as HIV testing and PrEP use among young sexual minority men who have been poorly reached through standard clinic services [17-19].

In prior work, 2 distinct apps, MyChoices (Keymind) and LYNX (APT Mobility), each designed to increase HIV testing and PrEP Initiation among young sexual minority men, were developed by separate research teams using different theoretical models to effect behavior change and different approaches for engagement (Multimedia Appendix 1). The process for development and pilot testing of these apps has been described previously [22-25]. In brief, both apps were developed with extensive formative research with young sexual minority men, including interviews and focus groups, beta testing, and pilot randomized controlled trials (RCTs). In parallel pilot RCTs conducted as part of the National Institutes of Health’s Adolescent Medicine Trials Network (ATN) for HIV Interventions, both MyChoices and LYNX were found to be feasible and acceptable, meeting predetermined go or no-go criteria for this current full-scale efficacy trial.

This study presents the results of a 3-arm, RCT conducted in 9 US cities in order to assess the efficacy of the LYNX and MyChoices apps to improve HIV testing and PrEP initiation among young sexual minority men.

## Methods

### Trial Design

This was a 3-arm, RCT with a 1:1:1 allocation ratio conducted as part of the ATN and the University of North Carolina/Emory Center for Innovative Technology (iTech), and implemented between October 2019 and May 2022 [26,27]. Consented participants completed a baseline assessment and follow-up assessments at 3- and 6-months post baseline. Some participants also completed an additional follow-up assessment between 6 and 12 months post baseline (see Adjustments in Trial Design in Response to the COVID-19 Pandemic section for more details).

### Participants

To be eligible participants had to (1) identify as cisgender men, (2) be aged 15-29 years, (3) self-report being HIV serostatus negative or HIV serostatus unknown at screening, (4) not have had an HIV test in the past 3 months, (5) not be currently taking PrEP, (6) self-report at least 1 episode of anal intercourse with a male or transgender female partner during the last 12 months, (7) had to own or have regular and ongoing access to an iOS or Android smartphone, (8) be willing and able to download the MyChoices or LYNX app, and (9) be able to understand, read, and speak English.

Participants were recruited through social media, flyers, and direct outreach at local venues and clinical sites across 9 sites: Atlanta, Georgia (study site: PRISM Health); Boston, Massachusetts (study site: Fenway Health); Bronx, New York City (study site: the Adolescent AIDS Program at Montefiore); Chapel Hill, North Carolina (study site: University of North Carolina, Chapel Hill); Charlotte, North Carolina (study site: RAIN); Chicago, Illinois (study site: AYAR at CORE Center); Houston, Texas (study site: Texas Children’s Hospital); Philadelphia, Pennsylvania (study site: Children’s Hospital of Philadelphia); and Tampa, Florida (study site: University of South Florida).

### Ethical Considerations

The study procedures were reviewed and approved by the University of North Carolina institutional review board (IRB) as a single IRB-of-record (study 19-0260). IRB authorization agreements with all participating research entities were enacted. A waiver of parental consent was obtained for individuals aged 15-17 years. All enrolled participants completed a site-specific consent or assent, which contained the essential elements, including a description of the purpose, study procedures, and risks and benefits. No social harms or adverse events were reported. Compensation for completing the survey was set by each site in accordance with norms, cost of living, and input from their IRBs. Participants were not compensated for using the app. The protocol for ATN 143: COMPARE (Comparison of Men’s Prevention Apps to Research Efficacy) is registered at ClinicalTrials.gov (NCT03965221).

### Randomization and Masking

Randomization was computer generated and was equally distributed 1:1:1 for study arms. Randomization was stratified by site and participant age (15-18 and 19-29 years). This trial did not use masking. Following randomization, study staff supported participants randomized to the experimental arms through downloading the app, and then provided a brief, structured demonstration of the main components of the app.

### Interventions

#### Standard of Care

Participants in all study conditions received CDC fact sheets on HIV testing and PrEP Initiation and had access to any services available as standard of care (SOC) at each study site [28,29].

#### Experimental Condition: MyChoices Mobile App

**Figure 1:**
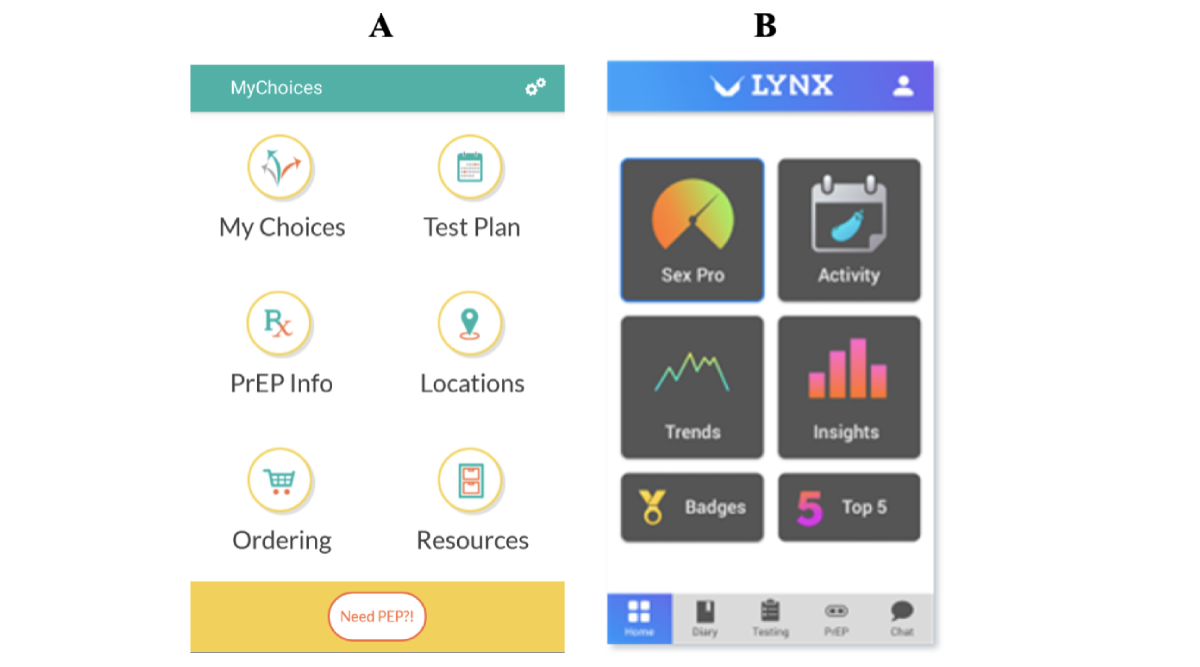
(A) MyChoices and (B) LYNX app landing pages.

As described and detailed previously [23,24], MyChoices was adapted, through extensive formative research and pilot testing, from an app previously demonstrated to increase HIV testing and PrEP Initiation in adult sexual minority men [22-24,30-32]. The MyChoices app is informed by the social cognitive theory [33] and includes the following primary components: sexual health information using a variety of formats (eg, infographics, videos, weblinks to educational resources); access to free home HIV and STI testing kits as well as condoms and lubricants; individualized, tailored HIV prevention recommendations and HIV testing plans based on monthly check-ins; reminders for HIV testing with geolocation-based notifications when near HIV testing sites and GPS-enabled maps with local HIV and STI testing locations and PrEP providers (Figure 1A).

#### Experimental Condition: LYNX Mobile App

As described previously [25], LYNX was developed through iterative formative research and pilot testing with young sexual minority men. The LYNX app is guided by the information-motivation-behavioral skills (IMB) model [34] and includes the following primary components: Sexual Health Promotion (Sex Pro) score that provides a personalized HIV risk score; sexual diary to help participants track sexual partners and encounters; reminders for HIV or STI testing; ability to order free home HIV and STI testing kits as well as condoms and lubricant; a geolocation map of HIV or STI testing sites and PrEP clinics; a PrEP information page including a roadmap on how to get on PrEP; a “trends and insights” page including infographics summarizing partner types and sexual activities; and a 2-way chat feature to answer questions and provide real-time assistance with PrEP navigation (Figure 1B).

### Privacy

To enhance confidentiality, in order to access either app, users initially entered their email address and a registration key (supplied following randomization) and set up a user-generated password. On subsequent visits to the app, users were required to supply their username and password or create a PIN. The apps timed out every time users left the app or when the phone went into sleep mode.

### Adjustments in Trial Design in Response to the COVID-19 Pandemic

Enrollment for the COMPARE study began in October 2019. At this point, all enrollment visits were conducted in person at the enrolling sites (n=83). All sites paused enrollment due to COVID-19 from March 2020 to June 2020. In June 2020, sites began to resume enrollment, with all enrollment visits occurring via videoconferencing. This continued until November 2021 when enrollment closed. Given the entirely remote procedures, in October 2021 and November 2021, enrollment was expanded to the entire United States. Participants were assigned to the study site that was closest to their zip code of residence. See Multimedia Appendix 2 for the enrollment timeline. In addition to changes in enrollment procedures, the COVID-19 pandemic significantly slowed down enrollment. In response, in collaboration with the ATN-wide Study Monitoring Committee, the decision was made to extend the enrollment period and shorten the full follow-up period for participants enrolled between June 2021 and November 2022 (follow-up ranging from 6 months to 11 months; n=40 participants were only eligible for a 6-month visit because they were enrolled in November 2022; the remaining 341 participants were eligible to complete an additional follow-up survey between 7 and 12 months post baseline). As such, our statistical analysis plan (also approved by the Study Monitoring Committee and IRB) was revised to shift the primary endpoint to be measured at the 6-month follow-up visit (rather than the 12-month follow-up visit). Importantly, based on eligibility criteria, all participants would be recommended to have an HIV test every 3 to 6 months according to CDC guidelines; as such, the outcome time frame remained clinically meaningful.

### Assessments

Baseline assessments were conducted at the enrolling study site or via videoconferencing and self-administered using a secure web-based data entry system. All follow-up assessments were self-administered and conducted off-site through the same secure web-based system. Follow-up assessments occurred at 6-month postbaseline for all participants, and an additional follow-up assessment occurred between 7- and 12-month post baseline for all participants enrolled prior to November 2022.

#### Primary Efficacy Outcome Measures

HIV testing and PrEP initiation over study follow-up were assessed by self-report at each follow-up visit. Specifically, participants were asked “How many times have you had an HIV test in the past 3 months?” with response options: “I have not had an HIV test in the past 3 months, 1 time, 2 times, 3 times, and 4 or more times.” For PrEP, participants were asked “In the past 3 months, have you taken PrEP (pre-exposure prophylaxis) to reduce the chance of getting HIV?” with response options: “Yes, I am on PrEP right now,” “Yes, but I’m not on PrEP anymore,” and “No.”

#### Secondary Outcome Measures

Time-to-event outcomes. As secondary outcomes, we examined the time to first HIV test and time to PrEP initiation from the baseline assessment using all available follow-ups. At each assessment, participants were asked the month and year of all their HIV tests and PrEP Initiation (if applicable) since the previous assessment. Since the specific dates when HIV tests and PrEP initiation took place were not assessed, we considered these events to happen on the last day of the month in which they reported the events.

#### HIV Testing Frequency

Participants were asked the number of times they were tested for HIV in the past 3 months (at 3- and 6-month visits) and 6 months (at 12-month visits). Responses were summed to obtain the total number of times participants were tested for HIV through the 12 months of follow-up, which ranged from 0 to 4 or more times.

#### PrEP IMB Scale

We created a 5-item scale to assess the 3 domains of the IMB model related to PrEP uptake: information (1 item), motivation (1 item), and behavioral skills (3 items). Sample items included, “I understand whether PrEP would be a good fit for me,” “I feel motivated to get on PrEP,” and “I know how to get PrEP”. Responses on a 5-point scale were averaged to reach a total IMB score. The Cronbach α=0.83 indicated strong internal consistency.

#### PrEP Self-Efficacy

We adopted the PrEP Adherence Self-Efficacy Scale [35] to assess PrEP self-efficacy in the past month with 10 items asking how confident participants had been that they can or could “integrate PrEP into your daily routine” or “take a PrEP pill every day even when you aren’t feeling well.” Response options ranged from 0=“could not do at all” to 10=“completely certain could do.” We obtained a total PrEP self-efficacy score based on the average of all 10 responses. The Cronbach α=0.96 indicated strong internal consistency.

#### PrEP Stigma

We assessed PrEP stigma with 12 items drafted by the study team, including “I can tell my friends that I am using PrEP,” “I would not want a sexual partner to see my PrEP pills in my medicine cabinet,” and “People would think I am sexually risky if they find out that I take PrEP.” Responses on a 5-point scale were reverse-coded as needed and averaged across 12 items such that higher scores indicated higher PrEP stigma. The Cronbach α=0.76 indicated moderate internal consistency.

#### Discussing PrEP With Health Care Provider

In order to measure the first steps in the PrEP care continuum, we assessed whether participants talked to a health care provider about PrEP (yes vs no).

#### PrEP Interest

Participants who had not initiated PrEP were asked about their interest in taking PrEP with a single item with response options ranging from 1=“not at all interested” to 5=“extremely interested.”

#### App Use Measures

We assessed the overall number of log-ins in each of the apps, the number of calendar days with app logins, and the total amount of time spent in the apps (minutes).

#### Sociodemographic Measure

To characterize the sample and examine potential moderating effects, we collected participants’ age, race and Hispanic or Latinx ethnicity, current relationship status (ie, single vs not single), and zip code of residence (which was aggregated to census regions: South, Northeast, Midwest, and West). We also assessed recent history of condomless anal sex by asking participants a series of questions about past-3-month sex partners, type of sex with those partners, and condom use during sex [36,37].

### Power and Sample Size

Using data from our pilot study, we powered the study to see a significant difference between each experimental condition compared to the standard of care assuming a baseline past-year HIV testing rate of 40%, HIV testing rate in the interventions arm at 77% (risk ratio [RR] 1.56), any app use in intervention arms of 60%, α=.05, and power=.80. With 70% retention at 12 months follow-up and equal allocation, we estimated a sample size of 450 (n=150 per arm, 33%). Notably, in June 2021, at the request of the ATN-wide Study Monitoring Committee, we reran our sample size calculation with all of the same assumptions but based on the higher-than-expected retention demonstrated at that point in the study (approximately 90% at each visit). The new targeted sample size was 351 (n=117 per arm, 33%).

### Statistical Analysis

Means for continuous variables and frequencies for categorical variables were calculated to describe the participants at baseline, overall, and stratified by intervention condition to assess balance in baseline characteristics.

An intent-to-treat approach was used, analyzing participants in the study arm to which they were assigned. As missing data was minimal (less than 10%), we used complete case analysis for each study aim. The primary efficacy analyses compared HIV testing and PrEP Initiation between the study arms over the study follow-up (ie, 6 months) using chi-square tests. Risk ratios and their corresponding 95% CIs were also calculated using log-binomial regression. Moreover, we examined differences between the study arms in the hypothesized mediators, including information, motivation, and behavioral skills related to PrEP Initiation, PrEP self-efficacy, PrEP stigma, discussing PrEP with a health care provider, and PrEP interest.

Secondary outcomes included time-to-event analyses for HIV testing and PrEP Initiation through the 12 months of follow-up using Kaplan-Meier survival curves. We examined overall differences across study arms using log-rank tests, and Cox proportional hazard models to obtain hazard ratios by study arm. We also examined the frequency of HIV testing over 12 months using Poisson regression models with robust standard errors.

We also assessed whether the intervention effect on the outcomes differed by baseline characteristics (ie, age, race or ethnicity, region, condomless anal sex, relationship status, depression and anxiety, and prepandemic enrollment). These moderator analyses were chosen a priori (with the exception of the pandemic measure) and were evaluated with interaction terms; we probed potential moderation in post hoc stratified analyses if the *P* value of interaction terms was less than .20.

In secondary analyses, we explored dose-response relationships by examining the effect of measures of usage of each app on HIV testing and PrEP Initiation at 6 months using log-binomial regression. In all dose-response analyses, we only considered app usage until the date of the assessment when outcomes of interest (ie, HIV testing and PrEP Initiation) were first reported. Since app usage measures were right-skewed, we winsorized these variables at the 98th percentile in all regression analyses [38].

## Results

**Figure 2:**
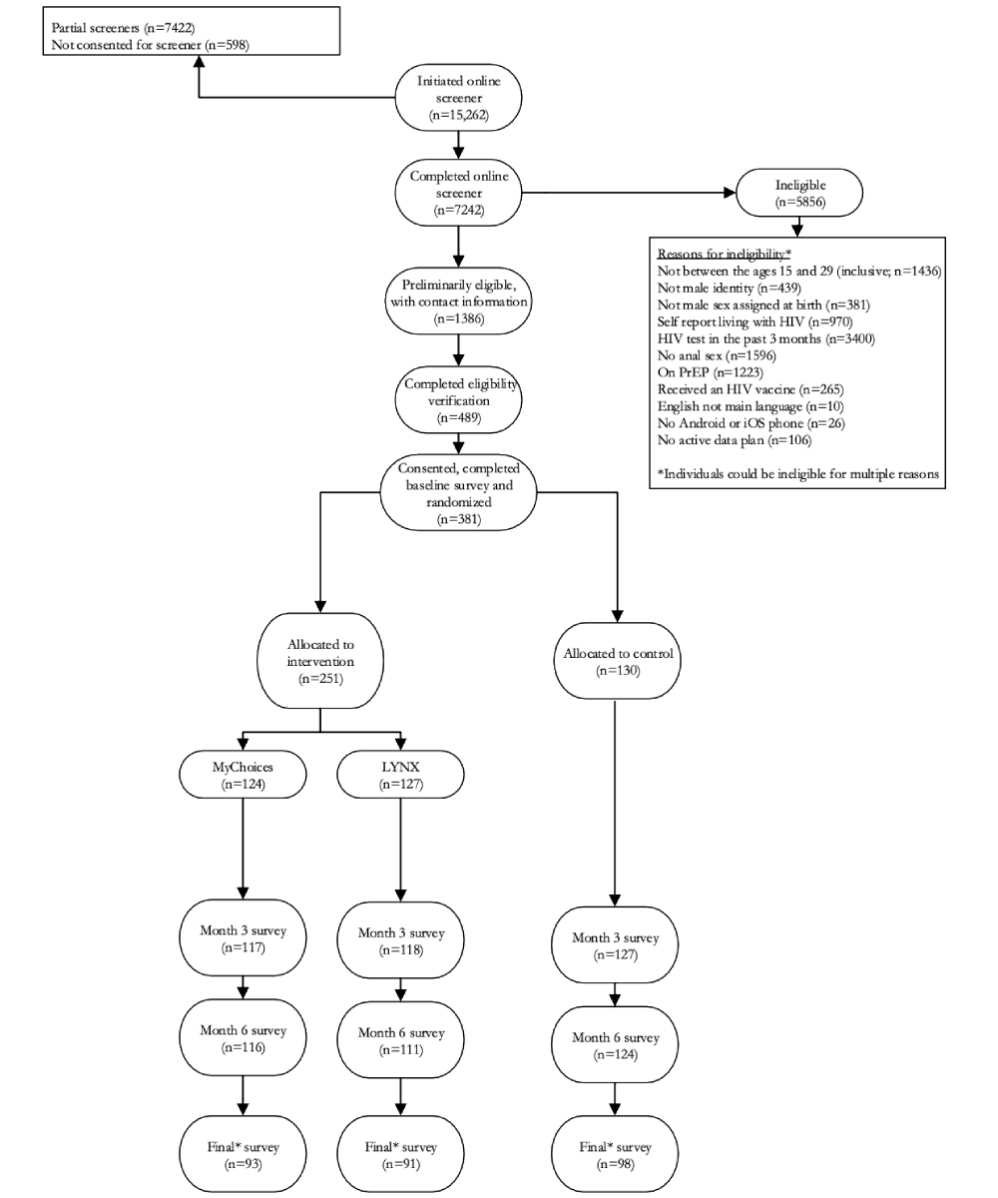
COMPARE (Comparison of Men's Prevention Apps to Research Efficacy) study CONSORT (Consolidated Standards of Reporting Trials) diagram, 2019-2022. PrEP: pre-exposure prophylaxis. *Final follow-up period ranged from 7-12 months postbaseline. Of the 381 enrolled, 341 were eligible to complete this final follow-up (n=111 in MyChoice; n=115 in LYNX; n=115 in standard of care).

Online eligibility screeners were completed by 7,242 individuals. Of those, 1,386 screened initially eligible and provided contact information. Common reasons for ineligibility included having a recent HIV test and not reporting recent anal sex. Four hundred and eighty-nine individuals were able to be contacted and attended the initial visit. Among these, 381 participants consented, completed a baseline assessment and were randomized to either the MyChoices app (n=124), the LYNX app (n=127) or the comparison condition (n=130). Among randomized participants, 95% completed a 3-month follow up assessment and 92% completed a 6-month follow up assessment. Figure 2  depicts participant flow throughout the study from screening to follow-up.

Sample characteristics at baseline are described in Table 1. The mean age was 22.4 (SD 3.2); 51% were White, non-Hispanic (n=194), 18% were Black, non-Hispanic (n=67), and 18% were Latino (n=67). 60% (n=228) identified as gay. 53% (n=200) resided in the Southern United States. At baseline, participants self-reported the following: 29% (n=110) had never had an HIV test, and 67% (n=253) had not had an HIV test in the prior 3 months. 85% (n=324) had never used PrEP, and an additional 13% (n=51) had not used PrEP in the prior 3 months. Sociodemographic and behavioral characteristics did not differ significantly by study arm.

**Table 1:** Baseline characteristics for the COMPARE^a^ study, overall and by study condition, 2019-2022.

			Total (N=381)		SOC^b^ (n=130)		LYNX (n=127)		MyChoices (n=124)
Age (years), mean (SD)			22.4 (3.17)		22.8 (3.34)		22.3 (3.20)		22.2 (2.96)
**Race and ethnicity, n (%)**									
	Asian, non-Hispanic	34 (9)		10 (7.8)		12 (9.5)		12 (9.8)	
	Black, non-Hispanic	67 (17.7)		25 (19.4)		26 (20.5)		16 (13)	
	Latino or Hispanic	67 (17.7)		29 (22.5)		23 (18.1)		15 (12.2)	
	White, non-Hispanic	194 (51.2)		58 (45)		59 (46.5)		77 (62.6)	
	Other or Mixed	17 (4.5)		7 (5.4)		7 (5.5)		3 (2.4)	
**Census region of current residence, n (%)**									
	Midwest	56 (14.7)		19 (14.6)		18 (14.2)		19 (15.3)	
	Northeast	112 (29.4)		36 (27.7)		39 (30.7)		37 (29.8)	
	South	200 (52.5)		68 (52.3)		67 (52.8)		65 (52.4)	
	West	13 (3.4)		7 (5.4)		3 (2.4)		3 (2.4)	
**Insurance status, n (%)**									
	Uninsured	43 (11.3)		12 (9.2)		12 (9.5)		19 (15.5)	
	Own insurance	136 (35.8)		46 (35.4)		47 (37)		43 (35)	
	Parent’s insurance	196 (51.6)		70 (53.9)		67 (52.8)		59 (48)	
	Do not know	5 (1.3)		2 (1.5)		1 (0.8)		2 (1.6)	
**Sexual orientation, n (%)**									
	Bisexual	69 (18.1)		23 (17.7)		23 (18.1)		23 (18.6)	
	Gay or homosexual	228 (59.8)		78 (60)		78 (61.4)		72 (58.1)	
	Queer or same sex or other	77 (20.2)		26 (20)		22 (17.3)		29 (23.4)	
	Heterosexual or straight	7 (1.8)		3 (2.3)		4 (3.2)		0 (0)	
**Relationship status, n (%)**									
	Single	182 (47.9)		62 (47.7)		61 (48.0)		59 (48)	
	Has boyfriend, girlfriend, partner, or lover	119 (31.3)		39 (30)		39 (30.7)		41 (33.3)	
	Casually dating	73 (19.2)		27 (20.8)		27 (21.3)		19 (15.5)	
	Married, civil union, or domestic partnership	6 (1.6)		2 (1.5)		0 (0)		4 (3.3)	
**Employment status, n (%)**									
	Unemployed	121 (31.9)		37 (28.7)		48 (37.8)		36 (29.3)	
	Employed part-time	126 (33.3)		41 (31.8)		40 (31.5)		45 (36.6)	
	Employed full-time	132 (34.8)		51 (39.5)		39 (30.7)		42 (34.2)	
**Student status, n (%)**									
	Currently enrolled	235 (61.8)		79 (60.8)		77 (60.6)		79 (64.2)	
	Not currently enrolled	145 (38.2)		51 (39.2)		50 (39.4)		44 (35.8)	
**Prior HIV test, n (%)**									
	Never	110 (29.1)		40 (30.8)		35 (27.9)		35 (28.7)	
	More than 3 months	253 (66.9)		87 (66.9)		85 (67.5)		81 (66.4)	
	Within past 3 months	15 (4.0)		3 (2.3)		6 (4.8)		6 (4.9)	
**Prior PrEP^c^ use, n (%)**									
	Never	324 (85.3)		112 (86.2)		110 (86.6)		102 (82.9)	
	More than 3 months	51 (13.4)		15 (11.5)		17 (13.4)		19 (15.5)	
	Within past 3 months	5 (1.3)		3 (2.3)		0 (0)		2 (1.6)	
**Condomless anal or vaginal sex (past 3 months), n (%)**									
	Yes	150 (39.4)		56 (43.1)		52 (40.9)		42 (33.9)	
	No	231 (60.6)		74 (56.9)		75 (59.1)		82 (66.1)	
**Enrolled Pre–COVID-19 shutdown, n (%)**									
	Yes	82 (21.5)		27 (20.8)		30 (23.6)		25 (20.2)	
	No	299 (78.5)		103 (79.2)		96 (76.4)		98 (79.8)	

^a^COMPARE: Comparison of Men's Prevention Apps to Research Efficacy.

^b^SOC: standard of care.

^c^PrEP: pre-exposure prophylaxis.

### HIV Testing

Compared to SOC (n=72, 59%), participants randomized to MyChoices were significantly more likely to have received at least 1 HIV test over 6 months of follow-up (n=87, 74%; *P*=.010; Table 2). Participants randomized to LYNX also had a higher proportion of testing but it did not meet the a priori threshold for statistical significance (n=80, 70%; *P*=.08). Individuals randomized to the MyChoices condition also reported more frequent HIV testing over follow-up compared to SOC (incidence rate ratio 1.25, 95% CI 1.01-1.54; *P*=.04), while this relationship did not reach significance for the LYNX condition compared to SOC (incidence rate ratio 1.13, 0.91-1.40; *P*=.26; Table 3). In time-to-event analyses (Table 4 and Multimedia Appendix 3), using all data (up to 12 months), those randomized to the MyChoices condition had a 45% higher likelihood (hazard ratio 1.45, 95% CI 1.07-1.95; *P*=.02) and those in the LYNX condition had a 34% higher likelihood (hazard ratio 1.34, 95% CI 1.00-1.81; *P*=.05) of receiving an HIV test compared to those in the SOC condition (log-rank *P*=.04).

**Table 2:** Estimated uptake of HIV testing and PrEP^a^ in the COMPARE^b^ study, 2019-2022.

	Any HIV test over 6 months				Any PrEP initiation over 6 months		
	n (%)	Risk ratio (95% CI)	*P* value	n (%)		Risk ratio (95% CI)	*P* value
SOC^c^ (reference)	72 (58.5)	1.0	—^d^	19 (15.6)		1.0	—
MyChoices	87 (74.4)	1.27 (1.06-1.53)	.01	23 (21.3)		1.37 (0.79-2.37)	.26
LYNX	80 (70)	1.19 (0.98-1.44)	.08	21 (19.6)		1.26 (0.72-2.21)	.42

^a^PrEP: pre-exposure prophylaxis.

^b^COMPARE: Comparison of Men's Prevention Apps to Research Efficacy.

^c^SOC: standard of care.

^d^Not applicable.

**Table 3:** Frequency of HIV testing in the COMPARE^a^ study, 2019-2022.

	Incidence rate ratio (95% CI)	*P* value
SOC^b^ (reference)	—^c^	—
MyChoices	1.25 (1.01-1.54)	.04
LYNX	1.13 (0.91-1.40)	.26

^a^COMPARE: Comparison of Men's Prevention Apps to Research Efficacy.

^b^SOC: standard of care.

^c^Not applicable.

**Table 4:** Time to first HIV test and PrEP^a^ initiation in the COMPARE^b^ study, 2019-2022.

	Time to first HIV test over 12 months		Time to PrEP initiation over 12 months	
	Hazard ratio (95% CI)	*P* value	Hazard ratio (95% CI)	*P* value
SOC^c^ (reference)	—^d^	—	—	—
MyChoices	1.45 (1.07-1.95)	.015	0.90 (0.47-1.71)	.75
LYNX	1.34 (1.00-1.81)	.052	1.17 (0.64-2.13)	.61

^a^PrEP: pre-exposure prophylaxis.

^b^COMPARE: Comparison of Men's Prevention Apps to Research Efficacy.

^c^SOC: standard of care.

^d^Not applicable.

According to the omnibus tests, the baseline characteristics did not significantly modify the effect of either app on HIV testing compared to SOC. However, in post hoc analyses (Table 5), both apps were significantly associated with HIV testing compared to SOC among White, non-Hispanic participants (MyChoices: RR 1.6, 95% CI 1.2-2.1; LYNX: RR 1.4, 95% CI 1.0-2.0); however, the conditions did not differ among non-White participants (MyChoices: RR 1.06, 95% CI 0.8-1.4; LYNX: RR 1.04, 95% CI 0.8-1.3). Among participants who reported engaging in condomless sex with a male or transgender female partner at baseline (Table 6), participants randomized to the MyChoices condition were nearly 6 times as likely to get an HIV test compared to SOC (RR 5.6, 95% CI 1.7-18.1; *P*=.004); the likelihood did not significantly differ among those not reporting recent condomless sex (RR 1.5, 95% CI 0.8-3.0; *P*=.21). Finally, participants who enrolled after the COVID-19 shutdown and were randomized to the MyChoices and LYNX conditions were significantly more likely to obtain an HIV test compared to the SOC condition (*P*=.005 and *P*=.02, respectively); the conditions did not differ for participants randomized prior to the COVID-19 shutdown (Table 7).

**Table 5:** Post hoc stratified analysis to assess moderation for HIV testing at 6 months by race or ethnicity in the COMPARE^a^ study, 2019-2022.

	White, non-Hispanic				Non-White		
	n (%)	RR^b^ (95% CI)	*P* value	n (%)		RR (95% CI)	*P* value
SOC^c^ (reference)	28 (48.3)	1.0	—^d^	43 (67.2)		1.0	—
MyChoices	56 (75.7)	1.57 (1.17-2.11)	.003	30 (71.4)		1.06 (0.82-1.37)	.64
LYNX	35 (68.6)	1.42 (0.67-3.00)	.03	45 (70.3)		1.05 (0.83-1.32)	.70

^a^COMPARE: Comparison of Men's Prevention Apps to Research Efficacy.

^b^RR: risk ratio.

^c^SOC: standard of care.

^d^Not applicable.

**Table 6:** Post hoc stratified analyses to assess moderation for HIV testing at 6 months by condomless anal or vaginal sex (past 3 months) in the COMPARE^a^ study, 2019-2022.

	Yes				No		
	n (%)	RR^b^ (95% CI)	*P* value	n (%)		RR (95% CI)	*P* value
SOC^c^ (reference)	33 (62.3)	1.0	—^d^	39 (55.7)		1.0	—
MyChoices	37 (90.2)	5.61 (1.74-18.09)	.004	50 (65.8)		1.53 (0.78-2.98)	.21
LYNX	36 (76.6)	1.98 (0.83-4.75)	.13	44 (64.7)		1.46 (0.73-2.89)	.28

^a^COMPARE: Comparison of Men's Prevention Apps to Research Efficacy.

^b^RR: risk ratio.

^c^SOC: standard of care.

^d^Not applicable.

**Table 7:** Post hoc stratified analyses to assess moderation for HIV testing at 6 months by pre–COVID-19 enrollment in the COMPARE^a^ study, 2019-2022.

	Yes				No		
	n (%)	RR^b^ (95% CI)	*P* value	n (%)		RR (95% CI)	*P* value
SOC^c^ (reference)	13 (68.4)	1.0	—^d^	59 (56.7)		1.0	—
MyChoices	13 (59.1)	1.04 (0.68-1.59)	.86	74 (77.9)		1.37 (1.13-1.68)	.005
LYNX	12 (54.6)	0.90 (0.57-1.40)	.63	68 (73.1)		1.29 (1.04-1.59)	.02

^a^COMPARE: Comparison of Men's Prevention Apps to Research Efficacy.

^b^RR: risk ratio.

^c^SOC: standard of care.

^d^Not applicable.

### PrEP Initiation

While participants in both MyChoices (21%) and LYNX (20%) arms had higher rates of initiating PrEP compared to SOC (16%), these differences were not statistically significant (*P*=.52; Table 2). The time-to-event analysis also did not demonstrate a significant difference in PrEP Initiation between study conditions (log-rank *P*=.72; Table 4 and Multimedia Appendix 3). Omnibus tests did not demonstrate an effect modification of PrEP Initiation by baseline characteristics or time of enrollment (all *P*>.10).

Given the null findings for PrEP Initiation, we explored, in post hoc analyses, whether the apps were efficacious in improving the hypothesized PrEP-related mediators. Participants randomized to the MyChoices condition had significantly higher scores for the PrEP IMB scale compared to the SOC condition (β=.24; 95% CI 0.03-0.44; *P*=.02). Among participants who had not initiated PrEP, participants randomized to the LYNX arm had a lower interest in PrEP compared to SOC (β=–.43; 95% CI –0.78 to –0.08; *P*=.02); PrEP interest among those randomized to the MyChoices arm who did not initiate PrEP was not significantly different from SOC (β=–.17, 95% CI –0.53 to 0.18; *P*=.33). The arms did not differ for PrEP self-efficacy, PrEP stigma scores, nor having spoken to a provider about PrEP.

### App Use

MyChoices and LYNX app use data are described in Table 4. In brief, for MyChoices, the mean number of log-ins was 8.55 (SD 17.0; range 1-186), and the mean total minutes on the app was 31.00 (SD 26.16; range 2.95-148.83). For LYNX, the mean number of log-ins was 12.43 (SD 13.10; range 1-74), and the mean total minutes on the app was 34.52 (SD 33.57; range 0.00-209.17). For either app, among those randomized to the experimental conditions, the number of app logins and time on the app were not significantly associated with HIV testing or PrEP Initiation (Table 8).

**Table 8:** Use and dose-response effects of MyChoices and LYNX apps on HIV testing and PrEP^a^ uptake at 6 months in the COMPARE^b^ study, 2019-2022.

		Range	Mean (SD)	HIV testing at 6 months		PrEP Initiation at 6 months	
				RR^c^ (95% CI)	*P* value	RR (95% CI)	*P* value
**LYNX (n=119)**							
	Number of log-ins	1-74	12.43 (13.10)	1.00 (0.98-1.02)	.97	0.97 (0.92-1.02)	.21
	Number of days with log-in to app	1-49	10.0 (9.78)	1.00 (0.98-1.02)	.98	0.94 (0.87-1.01)	.09
	Total minutes spent on app	<0.01-209.17	34.52 (33.57)	1.00 (0.99-1.01)	.72	1.00 (1.00-1.00)	.34
	Minutes per login	<0.01-13.39	3.35 (2.38)	0.97 (0.92-1.03)	.39	0.99 (0.98-1.01)	.37
**MyChoices (n=124)**							
	Number of log-ins	1-186	8.55 (16.96)	1.01 (0.99-1.04)	.29	0.94 (0.85-1.04)	.23
	Number of days with app log-in	1-153	7.58 (14.03)	1.02 (0.99-1.06)	.24	0.95 (0.85-1.06)	.35
	Total minutes spent on app	2.95-148.83	31.00 (26.16)	1.00 (1.00-1.00)	.44	0.99 (0.97-1.01)	.23
	Minutes per log-in	0.51-16.58	4.86 (3.04)	1.01 (0.97-1.04)	.72	0.97 (0.85-1.12)	.68

^a^PrEP: pre-exposure prophylaxis.

^b^COMPARE: Comparison of Men's Prevention Apps to Research Efficacy.

^c^RR: risk ratio

## Discussion

HIV remains a significant public health problem in the United States, with particular subgroups at increased risk, including young sexual minority men. mHealth interventions, delivered through mobile apps, have the potential to reach this population given their patterns of use. Routine HIV testing and PrEP use are 2 strategies that have been shown to be efficient and cost-effective at reducing HIV transmission, particularly among groups at higher risk for HIV [39,40]. However, barriers to widespread acceptance of these interventions among young sexual minority men have reduced their population-level effects. Our 2 community-driven and theory-informed apps, MyChoices and LYNX, increased the likelihood that a large, diverse sample of at-risk young sexual minority men across the United States received at least 1 HIV test over 6 months of follow-up compared to SOC; however, the apps did not statistically significantly improve the PrEP initiation. While these results were mixed, they are illuminating.

Both MyChoices and LYNX had a meaningful impact on HIV testing compared to SOC; though the effect size for MyChoices was slightly higher and was statistically significant. An approximately 25% relative increase in HIV testing among young sexual minority men, as seen in this study, is similar to those seen in the evidence-based interventions promoted by the CDC for HIV testing [41]. It is estimated that people living with HIV who are unaware of their serostatus account for up to 40% of new sexually transmitted HIV cases, and knowing one’s status reduces HIV sexual transmission by 71% [42]. Given the potential reach and successful implementation, these apps could have a substantial impact on the epidemic among young sexual minority men in the United States.

Over the course of the study follow-up, the amount of time that youths engaged with the apps was relatively modest. Future work should explore the role of different types of reminders for repeat testing and assess whether additional information (eg, about having more fulfilling sex, signs and symptoms of STI, other sexual health topics of interest) might lead to more sustained use, and greater impact in establishing regular testing patterns and PrEP use [43]. That said, notably, among young sexual minority men randomized to MyChoices and LYNX, the number of log-ins and the time spent on the app were not associated with each outcome, suggesting that it may not necessarily be the dose but the content and functionalities that matter the most. Future studies should examine the role of app engagement to examine the optimal balance between quantity versus quality [44].

Despite omnibus tests for interactions not showing evidence for effect modification by baseline characteristics, stratified analyses demonstrated that both apps were significantly associated with HIV testing compared to SOC among White, non-Hispanic participants; however, the conditions did not differ among non-White participants. The null finding among non-White participants may have been due to the lack of statistical power due to small sample sizes. However, given that young sexual minority men of color are at disproportionate risk of acquiring HIV, it is essential to further explore how to support HIV testing uptake in this population [45], and future iterations of these apps should attempt to address any gaps. On the other hand, the MyChoices app was substantially more effective in improving HIV testing among participants who had engaged in recent condomless sex with a male or transgender female partner, suggesting that this app is appropriately routing young sexual minority men who are at higher risk of HIV acquisition toward HIV testing.

These mHealth interventions and the format of the efficacy trial facilitated the continuation of this study during the COVID-19 pandemic. Even more, post hoc analyses suggested that individuals enrolled after the COVID-19 shutdown received the greatest benefit of both MyChoices and LYNX. It is possible that the availability of at-home HIV tests, condoms, and lubes through the apps was even more essential to isolate young sexual minority men during this time frame. Given the prediction that additional pandemics are increasingly more likely due to climate change and ease of global travel, this is an important finding. Additionally, it suggests that young sexual minority men who may be isolated for reasons other than pandemics (eg, living in rural areas) might further benefit from these apps and the resources that they provide [19].

MyChoices and LYNX demonstrated a small and nonsignificant impact on PrEP Initiation among young sexual minority men in this study, including among subgroups. Modeling studies have suggested that even a modest increase in PrEP Initiation could have meaningful impacts on HIV incidence [46]. Reasons for the small effect sizes (particularly in comparison to the larger impact on HIV testing) are not fully understood; however, barriers to PrEP Initiation are greater than those for HIV testing—it is less widely available, requires clinical appointments (vs HIV testing within community-based organizations or even at home), has more cost implications and is a medicine that must be taken regularly. In this study, for those who did not initiate PrEP by 6 months, the most common reasons reported include not having a perceived need (47.3%), costs (31.2%), worry about side effects (32.2%), and concern about long-term effects (30.1%). Future app enhancements should further address these barriers, including highlighting the potential benefits of PrEP, linkage to a PrEP navigator and telehealth services for PrEP, more diverse content on side effects, and linkage to programs that help defray the costs. Additionally, since these apps were developed, several different PrEP modalities have been approved, including event-driven oral medication and injectable PrEP that can be administered every 2 months. Future iterations of these apps will need to accommodate the opportunities and challenges for youths now that a menu of PrEP choices is available.

This large, randomized efficacy trial has a number of strengths, including methodological rigor, community engagement throughout the app development and study planning, and the diversity of participants in terms of race or ethnicity, geography, employment, and insurance status. However, there are also limitations to consider. First, due to the timing of this study, the enrollment of participants was slowed due to the COVID-19 pandemic. As a result, we had to extend our enrollment dates and shorten our follow-up for a subset of participants to 6 months instead of the preplanned 12 months. However, given the enrollment criteria limited the sample to young sexual minority men at higher risk of HIV and who had not had a recent HIV test, according to the CDC, each participant would be recommended to an HIV test at least every 6 months, and thus this shorter follow-up time frame remains meaningful. Additionally, our time-to-event analysis, where all available data was used, demonstrated a similar pattern of results. This study also relied on self-reported HIV testing and PrEP Initiation, which increases the likelihood of social desirability bias and overestimation of the impact of the apps on these practices. This potential bias was reduced by the use of self-administered, web-based follow-up assessments. Additionally, the alternative would have required participants to show proof of HIV testing and PrEP Initiation, and we anticipated that the increased chances of missing data would have led to increased selection bias.

In spite of these limitations, the findings of this study are compelling and merit future exploration. In summary, future studies should enhance the functionalities related to PrEP to increase the apps’ impact on this important outcome, as well as add content and functionalities related to emerging prevention technologies as they arise, including doxycycline prophylaxis for STI prevention. Additionally, given the 25% relative increase in HIV testing among young sexual minority men who used the MyChoices app and the potential for a large population-level effect, implementation studies that test and determine best practices for dissemination of this app to young sexual minority men across the United States are warranted [47].

## Appendix

Multimedia Appendix 1 Comparison of theoretical models, key components and user experiences for the LYNX and MyChoices apps.

Multimedia Appendix 2 Enrollment by month for the COMPARE (Comparison of Men’s Prevention Apps to Research Efficacy) study, 2019-2022. Key: Red solid line=COVID-19 Shutdown (March 15, 2020); Green dashed line=re-opening of initial sites for enrollment (July 2020); Green solid line=all sites re-opened for enrollment (November 2020); Gray dashed line=expanded to national enrollment (October 2021).

Multimedia Appendix 3 Time to first HIV test (a) and PrEP Initiation (b) in the COMPARE study, 2019-2022.

Multimedia Appendix 4 CONSORT eHEALTH checklist (V 1.6.1).

## Abbreviations

ATN: Adolescent Medicine Trials Network
CDC: Centers for Disease Control and Prevention
COMPARE: Comparison of Men’s Prevention Apps to Research Efficacy
IMB: information-motivation-behavioral skills
IRB: institutional review board
mHealth: mobile health
PrEP: pre-exposure prophylaxis
RCT: randomized controlled trial
SOC: standard of care
STI: sexually transmitted infection

## Data Availability

The datasets generated during and/or analyzed during this study are available in the National Institute of Child Health and Human Development's Data and Specimen Hub data repository.
