# Supplementary figures and images for: The Effects of MyChoices and LYNX Mobile Apps on HIV Testing and Pre-Exposure Prophylaxis Use by Young US Sexual Minority Men: Results From a National Randomized Controlled Trial
Source: JMIR Public Health Surveill. 2025 Feb 5;11:e63428. doi: 10.2196/63428 (PMC11840373; doi:10.2196/63428)

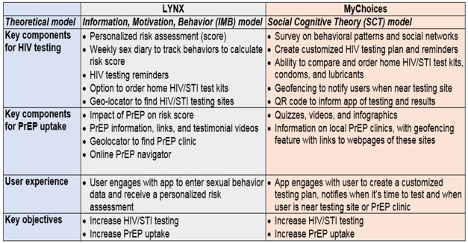

Supplement: Multimedia Appendix 1 [file publichealth_v11i1e63428_app1.png]

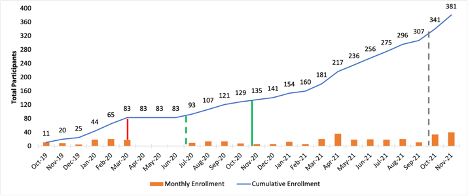

Supplement: Multimedia Appendix 2 [file publichealth_v11i1e63428_app2.png]

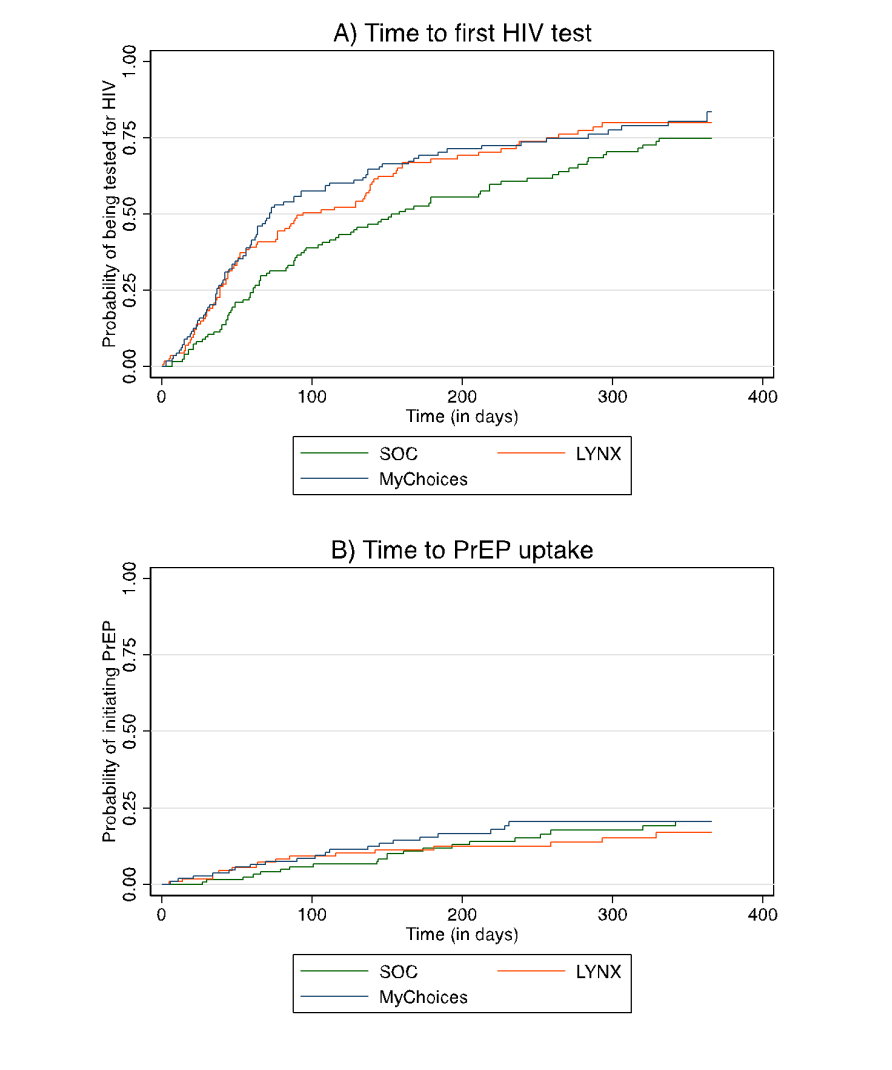

Supplement: Multimedia Appendix 3 [file publichealth_v11i1e63428_app3.png]
